# Dynamic facial trustworthiness perception in real-time social contexts

**DOI:** 10.3389/fpsyg.2025.1614643

**Published:** 2025-07-24

**Authors:** Haoming Qi, Dongcheng He

**Affiliations:** ^1^School of Information Network Security, People’s Public Security University of China, Beijing, China; ^2^Institute of Dataspace, Hefei Comprehensive National Science Center, Hefei, China; ^3^Herbert Wertheim School of Optometry and Vision Science, University of California, Berkeley, Berkeley, CA, United States

**Keywords:** trustworthiness, EEG, hemispheric asymmetry, social perception, affective cognition

## Abstract

**Introduction:**

Current understanding of the neural mechanisms underlying facial trustworthiness perception is primarily based on studies using static facial stimuli. However, real-life social interactions are dynamic and complex, and the neural processes involved in such naturalistic contexts remain largely unexplored.

**Methods:**

In this study, we analyzed EEG data collected by Chen et al. (2024) during a deception game involving two participants: a player and an observer engaged in real-time interaction. The player either followed instructions or made spontaneous decisions to lie or tell the truth, while the observer judged whether to trust the player based solely on his or her facial expressions. We examined observers’ behavioral data, event-related potentials, and interhemispheric EEG asymmetries in both signal magnitude and instantaneous phase.

**Results:**

The results revealed a significant effect of trustworthiness on hemispheric asymmetry in the observer’s centroparietal phase activities especially after ~800 ms post-stimulus until the end of the trial at 3,000 ms post-stimulus. Subsequent frequency-based analysis revealed that this asymmetry in phase progression was primarily driven by lateralized signal frequency.

**Discussion:**

These findings suggest that the perception of facial trustworthiness involves dynamic hemispheric lateralization. Whereas previous studies using static face stimuli indicate that trustworthiness perception occurs rapidly, our findings suggest that trustworthiness perception can be modulated by persistent and dynamic affective processing in real-time social contexts.

## 1 Introduction

A line of studies has demonstrated that people rapidly and intuitively form impressions of others’ social attributes, such as trustworthiness, based on facial expression perception. In a notable study, Willis and Todorov (2006) examined participants’ judgments of various traits (e.g., trustworthiness, attractiveness, etc.) from face stimuli presented for 100 ms, 500 ms, and 1,000 ms. They found that participants’ judgments were highly correlated across all time conditions, suggesting that reliable first impressions regarding the social attributes can be formed in as little as 100 ms. Building on this work, Todorov et al. (2009) conducted another experiment with a similar paradigm but employing finer-grained exposure durations ranging from 17 ms to unlimited viewing time. They reported that trustworthiness judgments were significantly above chance after only 33 ms of exposure. Subsequently, using a composite-face paradigm, that aligned trustworthy upper halves of faces and untrustworthy lower halves versus the opposite, Todorov et al. (2010) found that participants failed to discern between these two types of stimuli when judging trustworthiness with exposure times shorter than 100 ms. However, with longer viewing durations, participants rated the composites with trustworthy upper halves more positively, supporting the role of early holistic processing in trustworthiness perception. These studies demonstrated that impressions of social attributes formed from faces are instinctive evaluations, shaped more by perception than by deliberate reasoning. Such findings have been replicated by many subsequent studies (Bar et al., 2006; Porter et al., 2008; Holtz, 2015). Further investigations have explored additional factors that influence the perception of facial trustworthiness. For example, Krumhuber et al. (2007) found both genuine and fake smile faces were perceived to be more trustworthy than neutral faces, highlighting the role of emotional cues. Additionally, Robinson et al. (2014) identified the eyes and mouth as critical facial regions for facial trustworthiness perception, and demonstrated that manipulating the relative saliency of these regions could bias trustworthiness judgments. Other factors include the observer’s age (Cassidy et al., 2019), knowledge about others’ social category (Xie et al., 2019; Schmid et al., 2022), experience (Cheung et al., 2024), and even momentary associations (Fenske et al., 2005). These findings emphasize the roles of both perceptual and non-perceptual factors in biasing trustworthiness perception.

EEG has been instrumental in uncovering the temporal dynamics of cortical activities during facial trustworthiness perception, as well as its relationship to other cognitive functions. In an ERP study, Yang et al. (2011) observed a larger C1 component (within 40–90 ms post-stimulus) in response to trustworthy compared to untrustworthy faces. This finding supports previous behavioral findings that facial trustworthiness perception occurs at remarkably early stages. Additionally, a larger late positive component was detected in response to trustworthy faces, suggesting increased attentional demand. In another study, Calvo et al. (2018) investigated the interaction between emotional expression and trustworthiness perception. They presented faces with multiple types of emotional expression and found that facial expression processing could occur earlier than trustworthiness judgment, potentially reflecting a temporal hierarchy where emotional cues may precede and influence the evaluation of social attributes like trustworthiness. The effects of trustworthiness perception on face-related event-related potentials (ERPs) were also examined. N170 is a typical ERP signature, usually peaking between 140 and 230 ms, linked to the perception of emotional face-body compound stimuli (Meeren et al., 2005; Blau et al., 2007; Hinojosa et al., 2015). Previous studies have found that the trustworthiness of faces can modulate the subsequent component of N170, early posterior negativity (EPN), which suggested an affective evaluation process (Dzhelyova et al., 2012; Calvo et al., 2018). While these studies relied on explicit judgment tasks, other research has employed fast periodic visual stimulation (FPVS) to investigate implicit neural processing of facial trustworthiness. FPVS involves rapidly presenting visual stimuli at fixed frequencies, allowing the extraction of frequency-tagged EEG responses (Rossion, 2014). By updating face stimuli rapidly, this approach enables the decoding of EEG data patterns induced by relatively automatic and implicit neural functions, which are constrained by a short exposure duration. Using this approach, Swe et al. (2020) presented face stimuli at a base rate of 6 Hz, with trustworthiness systematically varying at 1 Hz. They successfully detected trustworthiness-related neural responses at 1 Hz, providing strong evidence for implicit encoding of facial trustworthiness perception. In another experiment using FPVS, trustworthy faces (oddballs) were interspersed every fifth stimulus among untrustworthy faces, and vice versa, resulting in an oddball rate of 1.2 Hz and a base rate of 6 Hz (Verosky et al., 2020). They found that the trustworthiness of oddball faces had a significant effect on the EEG signals at 1.2 Hz. These EEG studies align with behavioral data on the intuitive processing of facial trustworthiness, highlighting the role of implicit mechanisms in trustworthiness perception.

Regarding the cortical regions involved in facial trustworthiness perception, previous studies using fMRI have consistently identified the amygdala, a key region for affective processing, as showing different BOLD signals in response to face stimuli varying in perceived trustworthiness (Adolphs et al., 1998; Engell et al., 2007; Todorov and Engell, 2008; Said et al., 2009; Winston et al., 2013). It is well established that amygdala activity exhibits hemispheric asymmetry, particularly in the context of emotion processing (a review of neuroimaging studies regarding this issue: Baas et al., 2004). Early research using visual masking paradigms indicated that this lateralization depends on the observer’s awareness of emotional facial expressions, with unconscious processing primarily engaging the right amygdala (Morris et al., 1998). Additional theories about the functional differences between the left and right amygdala were also hypothesized, positing that the right amygdala supports rapid, automatic emotional responses, whereas the left amygdala is involved in more sustained and finer modulation of emotional arousal (Gläscher and Adolphs, 2003). Although the exact relationship between amygdala lateralization and EEG measures remains unclear, emotional processing is known to elicit hemispheric asymmetries in EEG signals. For example, the left frontal ERP was found to be lateralized to happy faces, while the right was lateralized to neutral faces (Graham and Cabeza, 2001). Such lateralization effects in EEG signals related to emotional valence were also detected in spectral analysis (Ahern and Schwartz, 1985; Pane et al., 2019) and instantaneous phase analysis (Costa et al., 2006; Val-Calvo et al., 2019; Cao et al., 2020). However, it is important to note that EEG is widely recognized for its limited spatial resolution, which restricts its ability to directly reflect subcortical activity within specific nuclei, such as the amygdala.

Previous research has significantly contributed to our knowledge about the cortical dynamics underlying facial trustworthiness perception and its associated mechanisms. However, the vast majority of these studies have relied on static facial photographs or algorithmically generated face images drawn from databases with preassigned trustworthiness scores. In contrast, real-world social interactions involve dynamic, temporally evolving facial expressions that unfold during interpersonal exchanges. Such naturalistic conditions may modulate trustworthiness perception over time in ways not captured by static stimuli. Yet perceiving actual faces in a real-time manner could complicate the cortical processing; however, little is known about the neural activities under such conditions. Particularly, the neural signatures revealing trustworthiness versus untrustworthiness in natural settings may not be readily inferred from previous findings based on static facial stimuli. To address this gap, we analyzed a novel EEG dataset collected simultaneously from a “player” and an “observer” during a task designed to encourage deception (data descriptor: Chen et al., 2024). At each time, the player decided to relay either the true or false information to the observer, who viewed the player’s face before choosing to trust the player or not. This design enabled us to investigate the effects of observers’ responses on their EEG activities, to uncover the EEG signatures reflecting the perception of trustworthiness in real-time social contexts.

## 2 Materials and methods

### 2.1 Description of the dataset

As illustrated by Chen et al. (2024), the dataset we used in the present investigation was curated from a data description study published in *Scientific Data.* This dataset contains a complete set of raw behavioral data and EEG data, as well as a preprocessed EEG dataset that were collected in a two-player deception experiment. Twenty-four participants (12 females, age: 25 ± 4.34 yrs) with normal or corrected-to-normal vision and no reported history of neurological disorder participated in this experiment for monetary rewards of around USD10/h. This experiment was approved by the Institutional Review Board with the number KUIRB-2019-0043-01. All participants were naïve to the experimental paradigm and gave written informed consent prior to the experiment.

### 2.2 Experimental procedures

**Figure 1:**
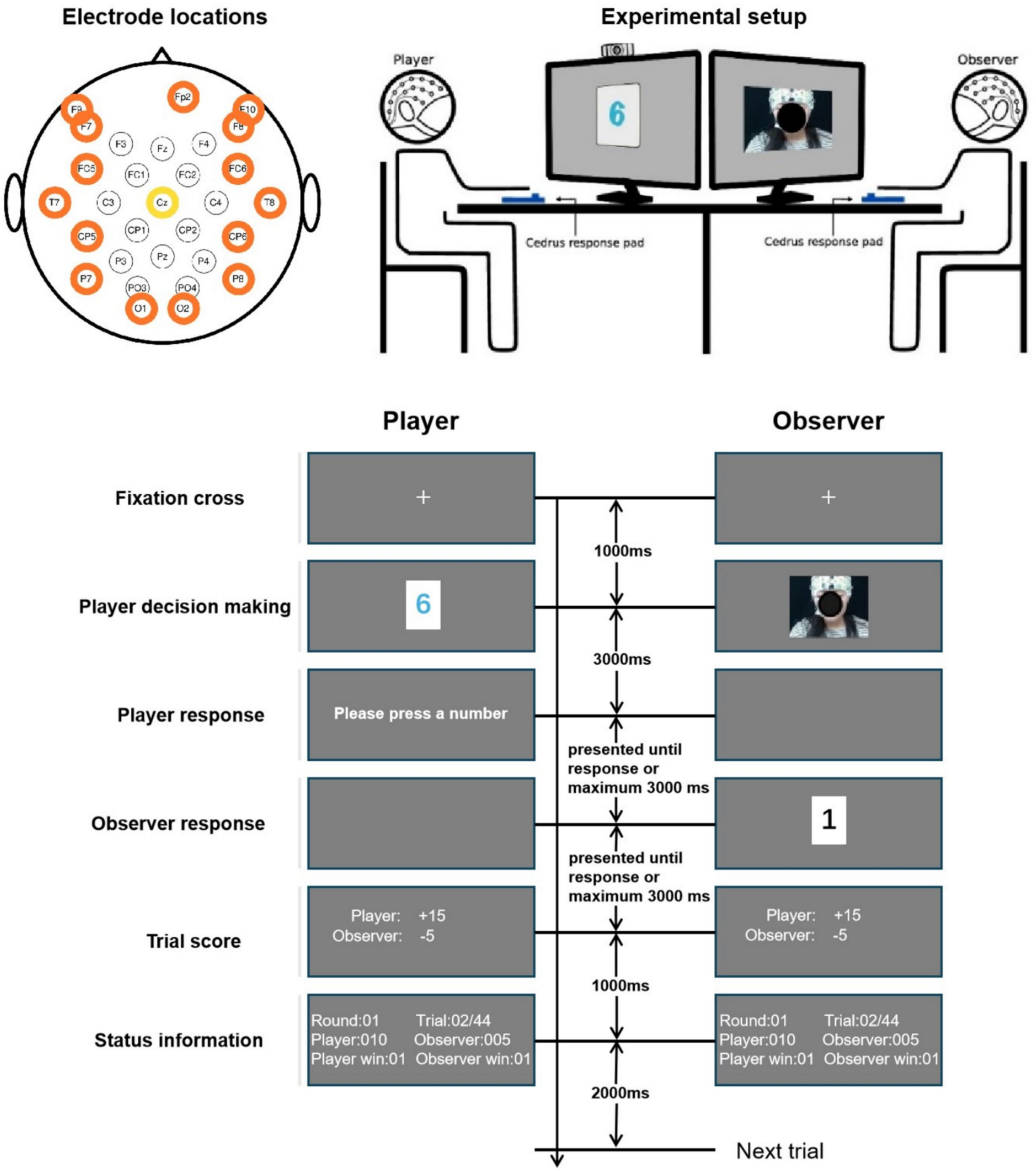
Illustration of the experiment. This figure was adapted from Figure 1 in Chen et al. (2024), and the colored circles around the electrode labels in this figure have no specific meanings.

As depicted in Figure 1, the task of this experiment involved two participants serving as the player and the observer, respectively, at each time. The two participants faced each other and sat in front of two separated 24-inch monitors (resolution: 1,920 × 1,080 ${\mathrm{px}}^{2}$ , refresh rate: 60 Hz; produced by LG, South Korea), with an HD pro C920 webcam (Logitech, Switzerland) installed on the top of the player’s monitor (Figure 1). As illustrated in Figure 1, a trial began with a fixation period of 1,000 ms, during which both participants were asked to fixate on a fixation cross that appeared in the center of their respective monitors. Then, a card containing a colored digit (ranging from 1 to 6) was presented on the monitor of the player’s side, whereas on the observer’s side, the monitor showed a live stream of the player’s face. This stage lasted for 3,000 ms, and during this period, the player had to decide which number to relay to the observer depending on the color (black, purple, or blue) of the presented digit. The three colors cued three different strategic conditions: (1) the instructed truth condition that the player had to relay the same number presented in the card to the observer; (2) the instructed lie condition that the player had to relay a different number than the presented one; and (3) the spontaneous condition that the player chose any number within the range of 1–6 to relay. Following this player decision stage, the player was asked to relay the number by pressing a corresponding button on a RB-740 response pad (Cedrus Corporation, United States) within 3,000 ms, during which only the background was presented on the observer’s side. Triggered by the player’s response, a card showing the relayed number in black was presented on the observer’s monitor and the observer was asked to decide whether the information was a lie (perceived untrustworthy) or the truth (perceived trustworthy) by pressing one of two buttons on a response pad within 3,000 ms. The observer would be the winner if he or she responded correctly, otherwise the player would be the winner; and the other party would be the loser. Upon the observer’s response, feedback was given to both participants according to a scoring system: if the player lied, the winner earned +15 points while the loser received −5 points; if the player told the truth, the winner gained +10 points and the loser still lost −5 points. This scoring system, explained before the experiment, was designed to encourage active participation of both participants by successfully deceiving and detecting in exchange for higher scores. At the end of each trial, a status screen displayed the cumulative score, number of trials and rounds won, and overall game progress. For the monitors of both sides, the trial scores that both participants acquired were presented for 1,000 ms and followed by a summary showing the accumulated scores, number of trials and rounds won by each side, as well as the progress of the experiment for 2,000 ms.

The 24 participants were separated into 12 pairs, and in each pair, two participants took turns serving as the player and the observer. This resulted in 24 paired cases, and 23 of them successfully finished the experiment. For each case, the entire experiment consisted of 11 sessions, in which each session contained 44 trials (spontaneous: 22, instructed lie: 11, instructed truth: 11) and these trials were presented in a randomly shuffled order. During all the sessions, EEG and responses of both participants were recorded. This experiment was programmed in Python using PsychoPy.

### 2.3 EEG apparatus

According to the data description, the EEG data was recorded with two BrainAmp amplifiers (Brain Products, Germany) from 30 EEG electrodes and an EOG electrode. Electrode locations on the caps are visualized in Figure 1. Due to connection issues, the Oz channel was removed from the dataset. The data was digitized at 500 Hz, nose-referenced online, and forehead grounded to the electrode Fpz. No online filtering was reported in the original data descriptor.

### 2.4 Data analysis

#### 2.4.1 Data preprocessing

In the preprocessed dataset, the data were down-sampled to 100 Hz and filtered at 1–49 Hz. Here an aggressive high-pass filter was used, with a cutoff frequency of 1 Hz. As reported by Acunzo et al. (2012), although a cut-off at 1 Hz has been widely used in the literature, a high-pass filter with a cut-off frequency higher than 0.1 Hz can alter the waveform of ERPs. On the other hand, a higher cut-off frequency can help mitigate slow baseline drift caused by body motion (Onikura et al., 2015). To replicate a real-life social context, this study did not use a chin rest or any other device to stabilize participants’ heads. Taking all these factors into account, we considered a 1 Hz cut-off acceptable. Artifacts were detected and rejected using EEGLAB functions and ICA-based algorithms with MATLAB (The MathWorks, United States). Data from each channel were then baseline corrected by 500 ms prior to the stimulus onset. Further details about the algorithms during preprocessing can be found in the data description (Chen et al., 2024). Since this study targeted facial trustworthiness, we selected the observers’ data from the “player decision making” stage (as labeled in Figure 1), which lasted 3,500 ms beginning from 500 ms prior to the facial expression display, and ending 3,000 ms afterwards. The resulting EEG signals were then re-referenced to the electrode Cz.

We conducted four analyses conditioned by the observers’ responses, either trustworthy or untrustworthy, toward the players. In these analyses, we analyzed the effects of the observer’s perception of trustworthiness by examining how their EEG data could reflect observers’ responses. First, we performed an ERP analysis to examine whether these social evaluations elicited amplitude differences across scalp electrodes and to identify the associated cortical regions. Second, to explore hemispheric asymmetries, we paired electrodes across the left and right hemispheres and compared the magnitude of their signals, assessing how perceptions of trustworthiness modulated these interhemispheric differences. Third, we extracted the instantaneous phases of EEG signals and repeated the interhemispheric analysis based on phase differences between homologous electrode pairs, enabling a finer-grained investigation of cortical synchrony and temporal alignment related to trustworthiness perception. A linear regression was performed on the phase progression-related profiles as a function of time to reflect the general frequency of the original signals. Finally, we calculated the weighted average of the frequencies of selected signals in a spectral centroid analysis to further examine how the instantaneous phase was affected.

Furthermore, as a control condition to the data segmentation based on trustworthiness, we randomly partitioned the data from each observer into two epochs 1,000 times with equal numbers of trials in each epoch that were balanced by the observer’s attitude to create a null distribution. The effects of trustworthiness on EEG signals were then compared to the null distribution in order to test the effects of trustworthiness against other factors.

#### 2.4.2 ERP analysis

For each observer, we segmented the EEG data into two conditions based on their responses: one consisting of trials in which the observer deemed the player as trustworthy, and the other consisting of trials in which the observer deemed the player untrustworthy. We first examined the behavioral and neural strategies by analyzing ERPs. Subsequently, we identified electrodes and time windows that exhibited signal differences associated with the observer’s responses. This was done by averaging ERP signals across all trials and participants separately.

#### 2.4.3 Interhemispheric electrode pairs

To examine interhemispheric differences, we paired electrodes across the frontal-posterior hemispheres, selecting those located at symmetric scalp sites. This resulted in 12 electrode pairs: F3–F4, F7–F8, F9–F10, FC1–FC2, FC5–FC6, C3–C4, CP1–CP2, CP5–CP6, P3–P4, P7–P8, PO3–PO4, and O1–O2. For each pair, we computed difference waveforms by subtracting the signal from the right hemisphere electrode from its left hemisphere counterpart. Consistent with the ERP analysis, EEG signals from each participant were divided into two conditions based on the observer’s responses. We then averaged the signals within each condition to generate signal profiles for statistical analysis.

#### 2.4.4 Instantaneous phase analysis

In this study, we performed an instantaneous phase analysis to uncover the dynamic spectral properties of EEG signals. Although Fourier transform has been widely used in spectral analysis, it is limited in analyzing nonstationary time-series as it is synthesized based on the entire signal and thus the time-varying features are averaged out, whereas instantaneous phase can reflect the dominant frequency at each time point with high temporal resolution by computing its derivative (Huang et al., 1998; Shea et al., 2021). Although time-frequency analysis reflects how spectral content evolves over time, it inherently averages signal components over a time window. As a result, its temporal resolution is limited by the window size or kernel used, making it less temporally precise than instantaneous phase analysis, which can offer finer time resolution.

In this regard, instantaneous phase analysis is suitable and has been widely used in synthesizing EEG signals related to brief mental processes (Schack et al., 1999; Kumar et al., 2018; Wang et al., 2006) or state transitions in rhythms (Freeman and Rogers, 2002; McIntosh and Sajda, 2020; Nelli et al., 2017). Here, we utilized this approach to track how the observers’ EEG signals varied dynamically and instantly in revealing the perception of trustworthiness.

The instantaneous phase dynamics of a signal was computed using the approach of Hilbert transformation, as illustrated by Equations 1 and 2.


(1) $$\hat{g}(t)=F^{-1}(-j\cdot \mathrm{sgn}(f)\cdot G(f))$$

(2) $$\phi_{g}(t)=\tan^{-1}(\frac{\hat{g}(t)}{g(t)})$$

where $\hat{g}(t)$ is the Hilbert transform of a given time-series g(t) , $F^{-1}$ is the inverse Fourier transform of the input signal, G(f) is the Fourier transform of g(t) , and $\phi_{g}(t)$ is the instantaneous phase of g(t) . We then unwrapped $\phi_{g}(t)$ to remove the discontinuity of the instantaneous phase profile and uncover the phase progression.

#### 2.4.5 Spectral centroid analysis

The spectral centroid of an EEG signal was computed using Equation 3 based on its power spectral density (PSD) ranging from 0 to 50 Hz along the frequency domain with a bin of 0.5 Hz.


(3) $$\mathrm{SC}=\frac{\sum_{k=0}^{N-1}f_{k}\cdot |X(k)|}{\sum_{k=0}^{N-1}|X(k)|}$$

where SC stands for spectral centroid, $f_{k}\;\text{and}|X(k)|$ are frequency and magnitude of PSD at bin *k*, and *N* stands for the total number of bins depending on the frequency range and bin size.

## 3 Results

### 3.1 Behavioral results

The mean (SD) accuracy across observers was 49.82% (2.74%), and a *t*-test revealed that the observers’ accuracy was not significantly different from the chance level of 50% (*t*(22) = −0.31, *p* = 0.76). The average number of trials in which observers deemed the player as trustworthy was 244.91 (10.63), while for untrustworthy it was 238.96 (10.64). A paired-samples *t*-test indicated no significant imbalance in the distribution of the two types of judgments (*t*(22) = 1.34, *p* = 0.19).

**Figure 2:**
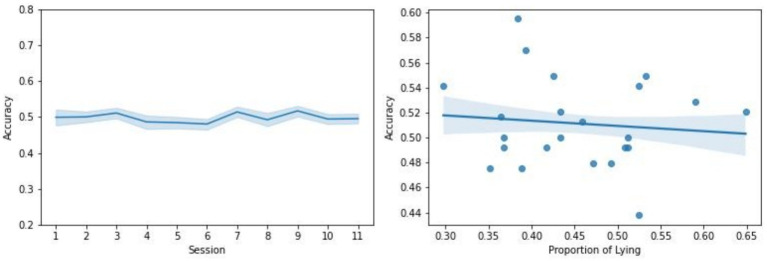
Left: Change in observers’ performance over the course of experiment. The mean accuracy across observers with respect to the 11 sessions of experiment is plotted. The shading indicates the standard error across observers. Right: Linear regression of observers’ performance with respect to the proportion of lying in the players’ behavior in the spontaneous tasks. Each dot represents a participant pair. The shading indicates the standard error of the estimate.

In addition, observers’ performance remained relatively stable throughout the experiment. As shown in the left panel of Figure 2, no significant difference in mean accuracy was found across the 11 sessions. A repeated-measures ANOVA with session number as the factor revealed no significant effect on mean accuracy (*F*(10, 220) = 0.55, *p* = 0.86, $\eta_{p}^{2}=0.02$). In the spontaneous condition, where players could freely choose to tell the truth or lie, we analyzed whether their behavior influenced observers’ responses through linear regression. As shown in the right panel of Figure 2, the proportion of lying ranged from approximately 30–65%. Despite this individual variability, there was no significant correlation between the observer’s mean accuracy and the player’s lying proportion (*r*(21) = −0.1, *p* = 0.65).

These findings suggest that observers’ judgments were largely intuitive and inaccurate, aligning with theories that social evaluations based on faces are instinctive and difficult without contextual information (see review by Todorov et al., 2015).

### 3.2 ERP analysis

**Figure 3:**
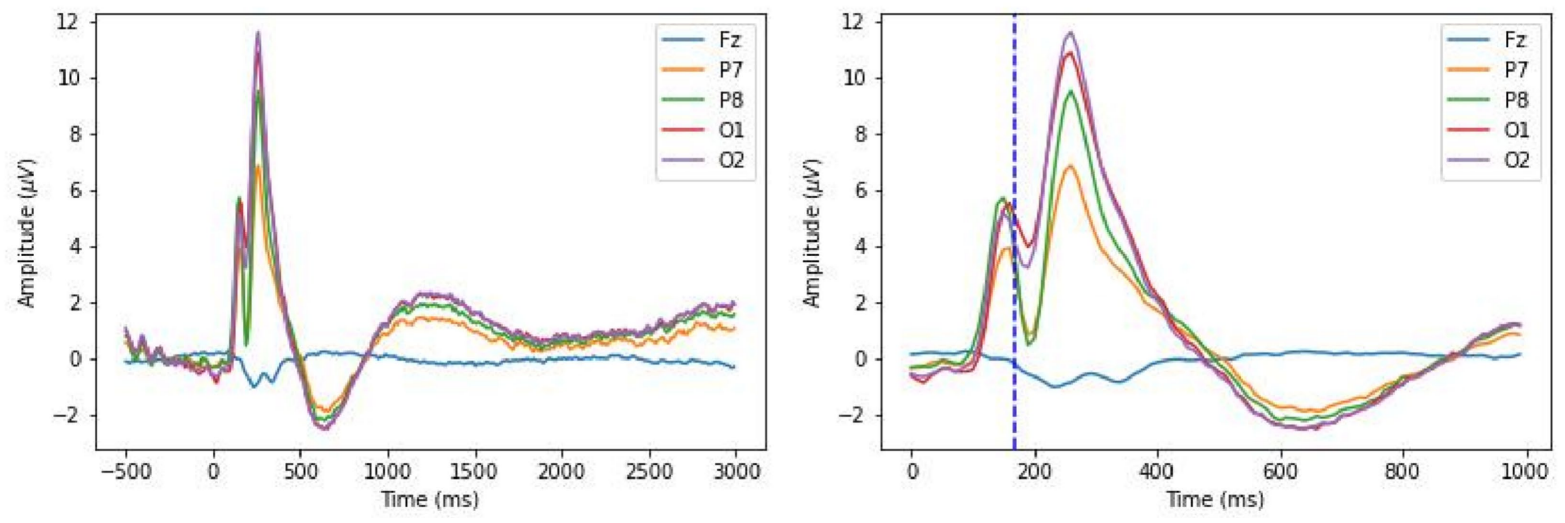
Grand average ERPs for the observers. The left panel shows the signals during the entire trial. The right panel shows the signals during 1,000 ms post-stimulus, and a dashed line marks 170 ms.

**Figure 4:**
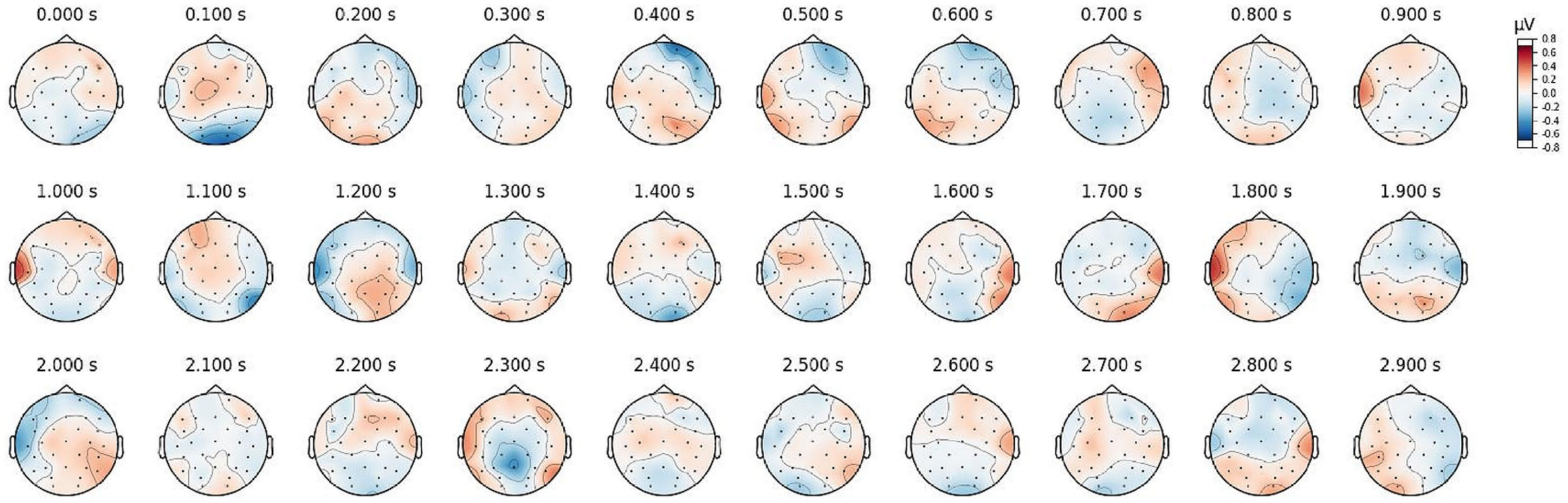
Topographics of grand ERP difference between trustworthy and untrustworthy epochs.

Figure 3 displays ERPs from selected frontal and posterior electrodes (Fz, P7, P8, O1, O2). A clear N170 component was observed around 200 ms, indicating engagement with facial expression processing. However, no significant effects of trustworthiness were found on ERP magnitudes. Figure 4 illustrates the topographic distribution of differences between trustworthy and untrustworthy trials, averaged over 100 ms windows. Across all electrodes, differences remained below 1 μV, and repeated-measures ANOVAs with trustworthiness as the main factor found no significant effects on observers’ ERPs during any time window ( α=0.05 ).

### 3.3 Interhemispheric comparisons

#### 3.3.1 Difference in signal magnitude

**Figure 5:**
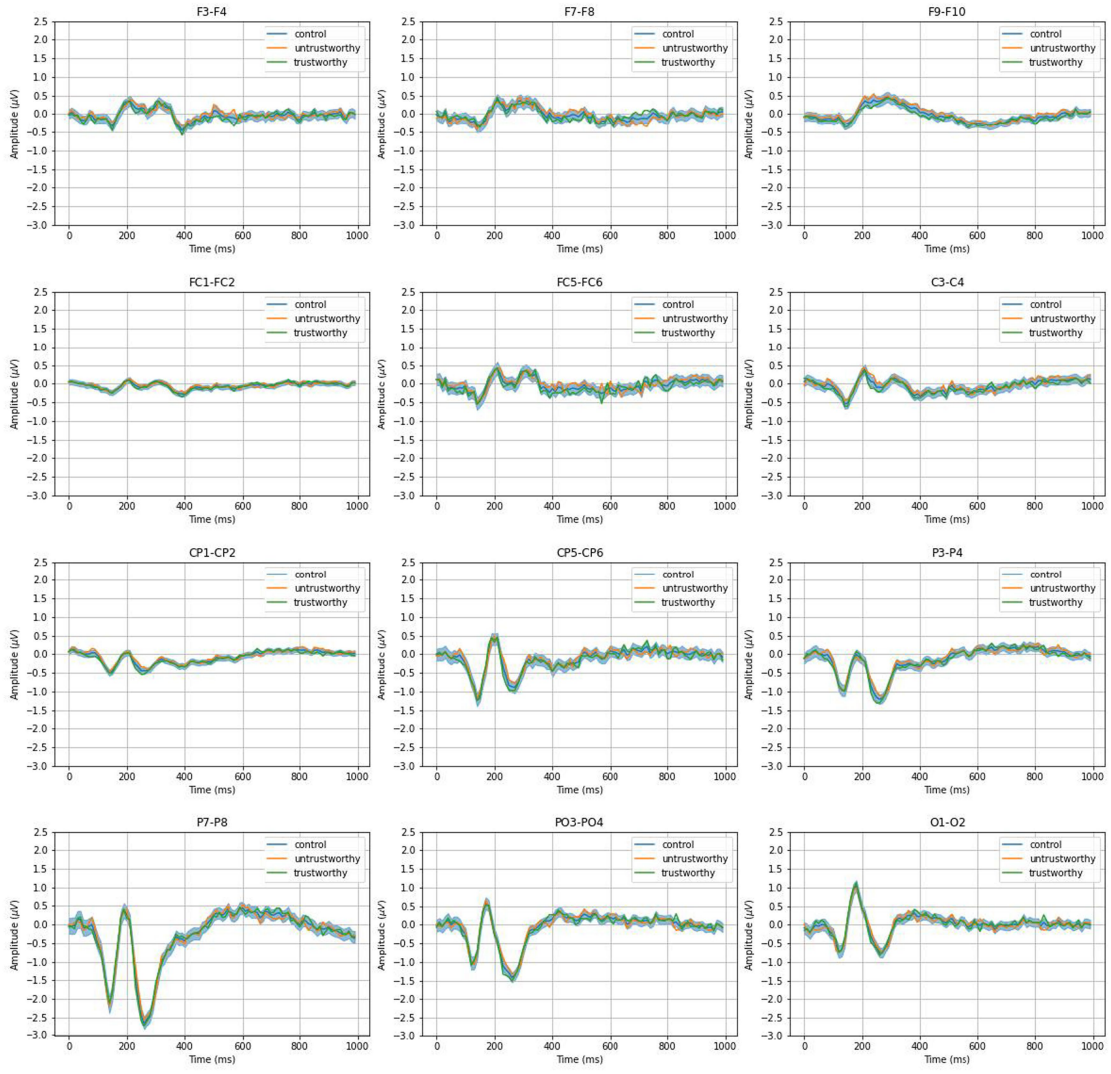
Grand average interhemispheric ERP differences as conditioned by trustworthy, untrustworthy, and control. The shading for the control condition indicates the 95% confidence interval of null distribution consisting of 1,000 times of resampling.

Figure 5 shows pairwise signal differences between left and right hemisphere electrodes, across trustworthy, untrustworthy, and control conditions. Notable differences were observed in P100 and P300 components, with stronger signals in the right hemisphere—especially evident at P7–P8, where subtracting P8 from P7 yielded negative peaks. For P100 (the average value during 80–130 ms), a paired-samples *t*-test detected a significant difference between the mean amplitude of P7 and that of P8 (*t*(22) = −3.32, *p* < 0.01). For P300 (250–300 ms): *t*(22) = −3.1, *p* < 0.01. A larger N170 component in the right hemisphere than that in the left hemisphere was also observed, mostly evident in O1-O2, where subtracting O2 from O1 yielded to a positive peak (170–220 ms: *t*(22) = 2.23, *p* < 0.05). These effects are also reflected in Figure 3. These effects confirm emotional face recognition and are consistent with prior reports of right-lateralized N170 components (Ibáñez et al., 2012; Nakajima et al., 2012; Gao et al., 2019; Dundas et al., 2013, 2014). Whereas right-lateralized N170 was only detected in response to face stimuli (Maurer et al., 2008; Rossion et al., 2003; Dundas et al., 2013, 2014), the effects of right lateralization on the other two peaks were reported for both word and face stimuli (Dundas et al., 2014).

However, comparisons between the trustworthiness conditions and control revealed no significant differences across electrode pairs. Profiles for both trustworthy and untrustworthy trials lay within the 95% confidence interval of the control condition in all cases.

#### 3.3.2 Difference in signal phase progression

In this section, we synthesized the phase progression of the signals from each electrode and calculated the phase shift between interhemispheric electrode pairs. To examine the effect of trustworthiness, for each observer, we determined the *conditioning interhemispheric phase shift*, by calculating the absolute difference in mean interhemispheric phase shift across trustworthy- and untrustworthy-related trials. We controlled it by the null distribution of that between two equal-size epochs for 1,000 times of random resampling, as mentioned in Section 2.4.1. Our null hypothesis was that the profiles of *conditioning interhemispheric phase shift* representing trustworthiness and random control should be equal if trustworthiness can induce no effect on the interhemispheric phase shift progression.

**Figure 6:**
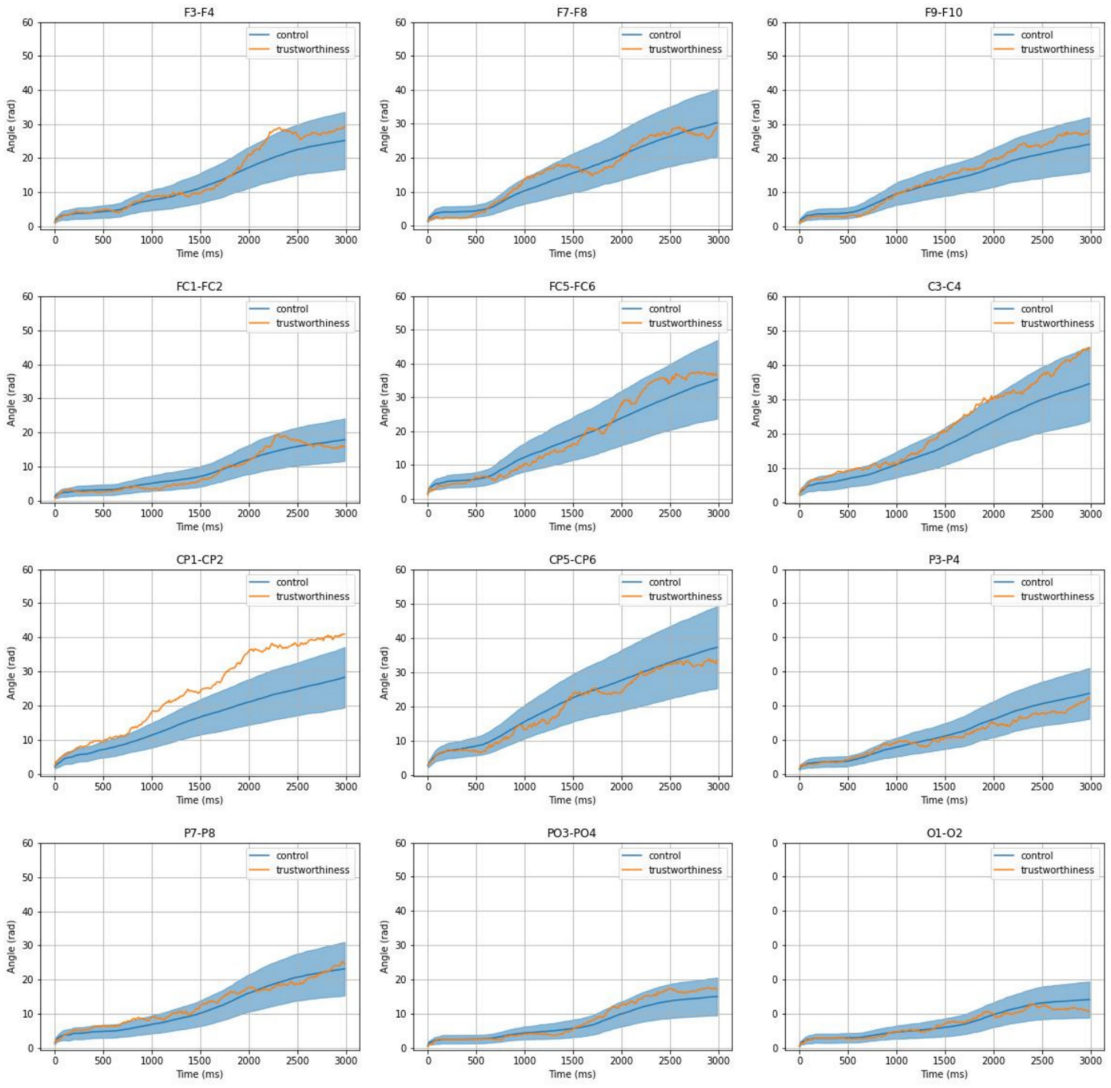
Mean profiles of conditioning interhemispheric phase shift on trustworthiness and control. The shading for the control condition indicates the 95% confidence interval of null distribution consisting of 1,000 times of resampling.

Averaging across all the observers, Figure 6 illustrates the effects of trustworthiness on interhemispheric phase shifts for all electrode pairs. We observed that phase dynamics were generally asymmetrical between the two hemispheres. Also, these effects appeared to be highly variable, as *conditioning interhemispheric phase shift* profiles on both conditions deviated from zero over time across electrode pairs.

Comparing *conditioning interhemispheric phase shift* profiles between the trustworthiness condition and the null distribution, significant phase asymmetries emerged in centroparietal regions—most notably at CP1-CP2—where the phase shift exceeded the 95% confidence interval of the control condition, particularly from ~800 ms to 3,000 ms post-stimulus. This suggests that trustworthiness perception enhances hemispheric divergence in phase progression.

**Figure 7:**
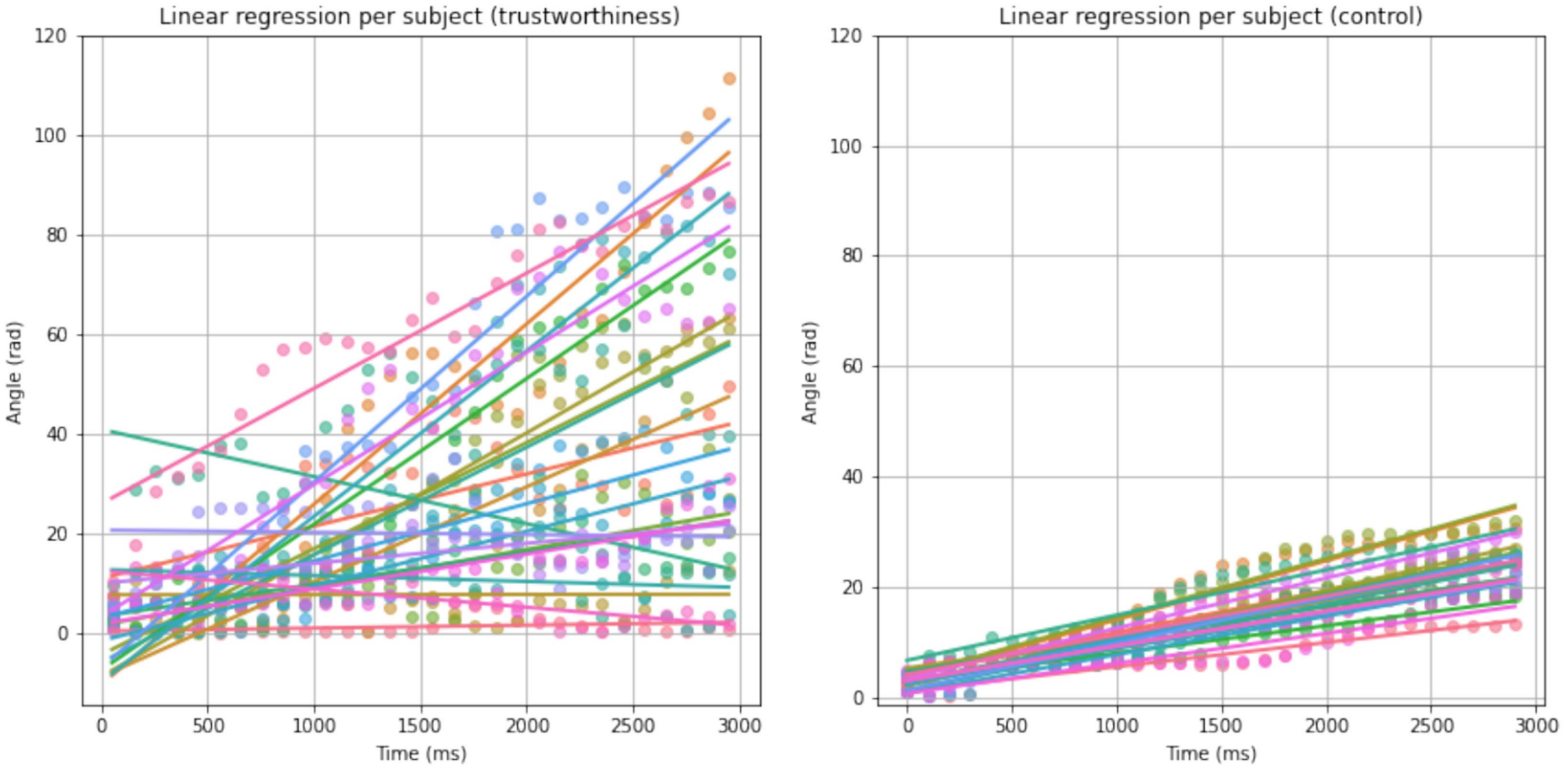
Results of linear regression on *conditioning interhemispheric phase shift* per subject for trustworthiness and control conditions. Each color represents an observer. For better visualization, the scatters are binned by a time window of 100 ms.

**Figure 8:**
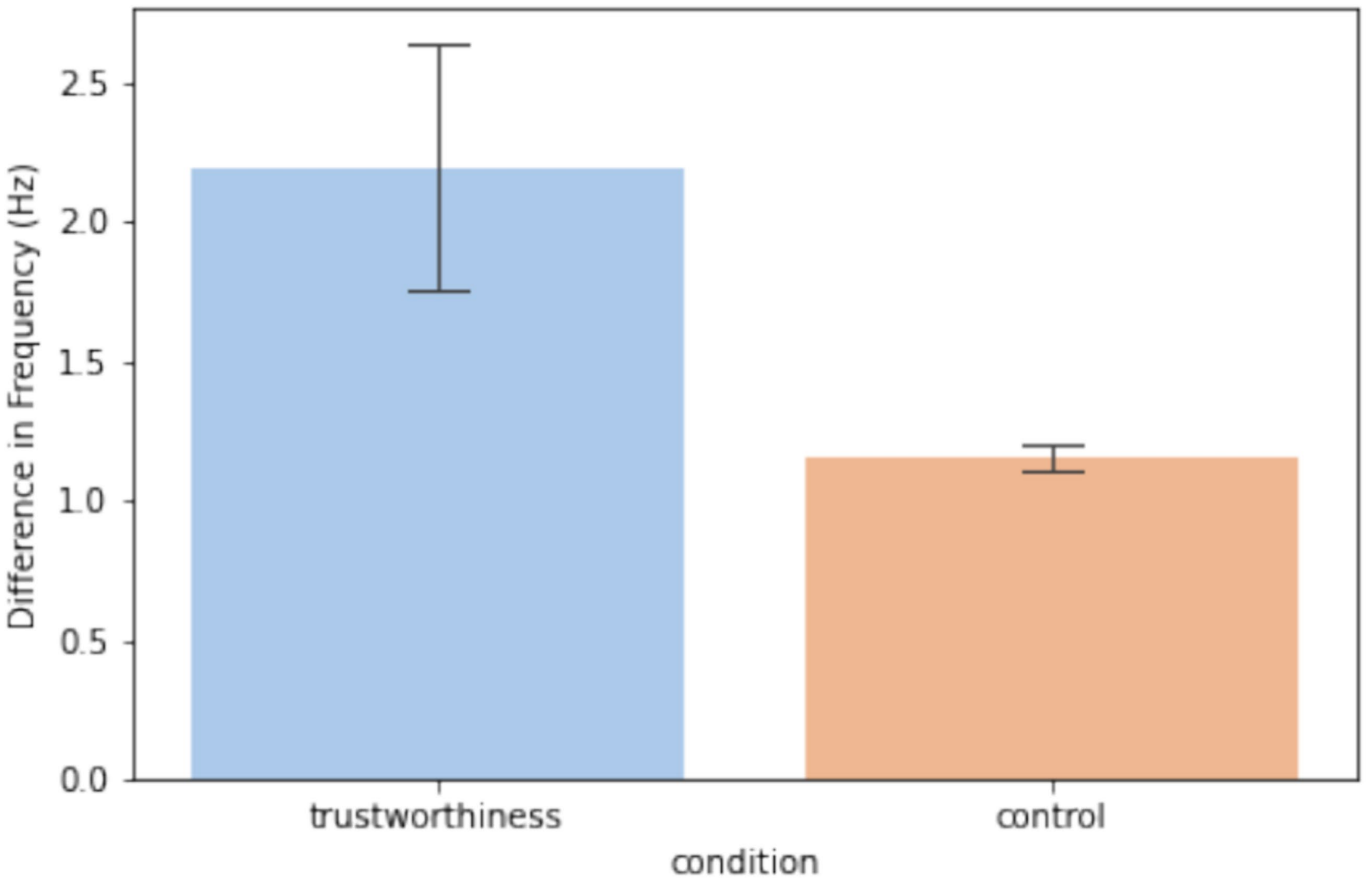
Slopes resulted by the linear regression analysis as transformed to be in a unit of Hz. The error bars indicate one standard error across observers.

From Equations 1 and 2, we know that the instantaneous phase is the integral of instantaneous frequency over time. We thereby performed linear regressions on each participant’s *conditioning interhemispheric phase shift* at CP1 - CP2 and analyzed the average difference in signal frequency between CP1 and CP2 based on the resulting slopes. As for the control condition, we used the median level within the null distribution for each observer, so that a repeated-measure comparison between trustworthiness and control conditions could be performed. Figure 7 shows the results of linear regressions and Figure 8 shows the slopes in a unit of Hz. The average frequency difference (slope) conditioned by trustworthiness and control were 2.18 ± 2.16 Hz and 1.16 ± 0.26 Hz, respectively. A repeated-measures ANOVA confirmed a significant difference between the trustworthiness and control conditions on the slopes (*F*(1, 22) = 5.91, *p* < 0.05, $\eta_{p}^{2}=0.21$). These results indicate a higher divergence between two hemispheres by means of signal frequency induced by facial trustworthiness perception than random states. More specifically, the frequency difference between signals in CP1 and CP2 was 1.16 Hz by average under random states, but perceiving trustworthy and untrustworthy faces induced a significantly increased interhemispheric divergence with an average degree of 2.18 Hz.

### 3.4 Spectral centroid analysis on CP1 - CP2

Visual inspection from the profiles of phase shifts in Figure 6 suggested a high slope difference between trustworthiness and control conditions during 800–2,000 ms. Based on these findings, we conducted spectral centroid analysis for CP1–CP2 using signals from 800 to 2000 ms. Mean spectral centroid values were compared across trustworthy and untrustworthy trials, and against the control condition, in which each time of random data segmentation resulted in two half-epochs, labeled as “control1” and “control2,” and averaged values across all times of resampling were used.

**Figure 9:**
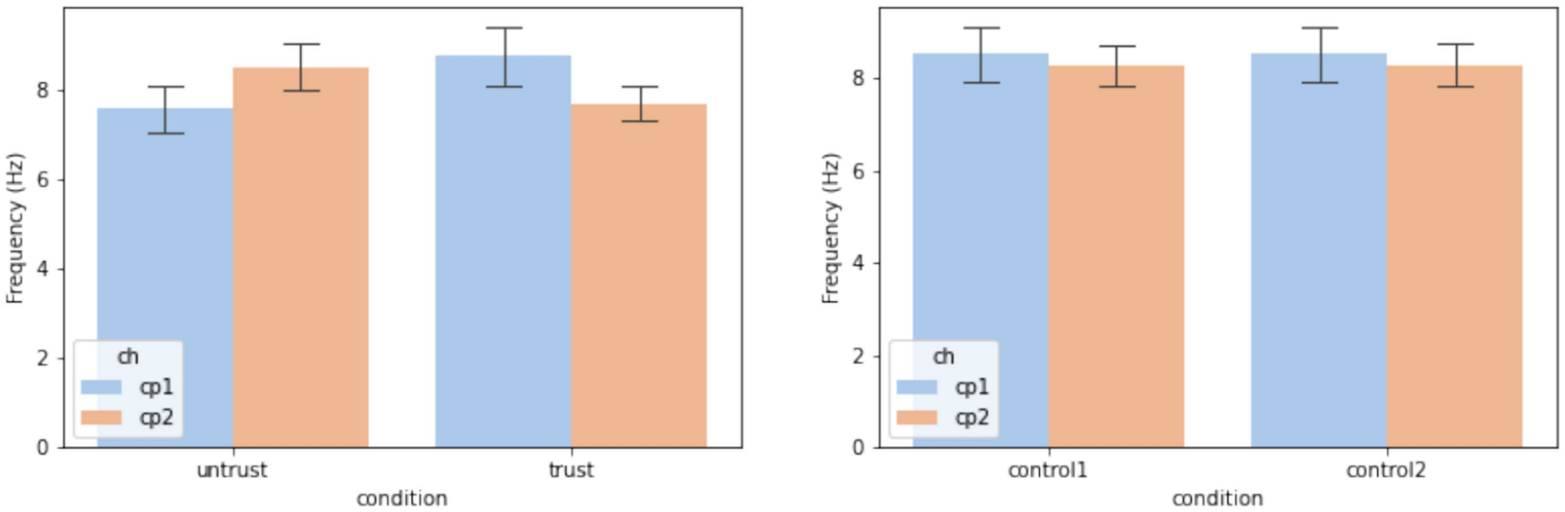
Spectral centroid of the signals from the electrode pair CP1 - CP2. The error bars indicate one standard error across observers.

As shown in Figure 9, we found that the spectral centroid at CP1 - CP2 was dependent on trustworthiness. A repeated-measures ANOVA with two factors (electrode and trustworthiness) revealed a significant interaction between the electrode and trustworthiness (*F*(1, 22) = 6.44, *p* = 0.018, $\eta_{p}^{2}=0.23$) on the mean spectral centroid, but did not detect any factorial effects (channel: *F*(1, 22) = 0.14, *p* = 0.71, $\eta_{p}^{2}<0.01$; trustworthiness: *F*(1, 22) = 0.03, *p* = 0.86, $\eta_{p}^{2}<0.01$). *Post hoc* analyses using Tukey’s HSD examining the simple effects of trustworthiness within the electrode revealed no significant differences between the trust and untrust conditions at either CP1 (mean difference = −1.18, 95% CI = [−2.96, 0.6], p-adjusted = 0.19) or CP2 (mean difference = 0.82, 95% CI = [−0.56, 2.19], p-adjusted = 0.24). However, the difference in the simple effects of trustworthiness across CP1 and CP2 was statistically significant, as indicated by a paired-samples *t*-test (*t*(22) = −2.54, *p* = 0.019, Cohen’s d = −0.67), confirming a crossover interaction pattern. Specifically, the spectral centroid was higher in trust condition than untrust at CP1, but this pattern reversed at CP2. In contrast, no significant effects were found in the control condition (channel: *F*(1, 22) = 0.37, *p* = 0.55, $\eta_{p}^{2}=0.02$; control: *F*(1, 22) = 0.1, *p* = 0.76, $\eta_{p}^{2}<0.01$; channel×control: *F*(1, 22) = 0.38, *p* = 0.54, $\eta_{p}^{2}=0.02$).

These results confirm the earlier findings, indicating that hemispheric asymmetry in the centroparietal region reflects trustworthiness perception. Specifically, the observed phase asymmetry appears to be driven by a lateralization of signal frequency: trustworthy faces were likely to induce relatively higher frequencies in the left hemisphere, while untrustworthy faces could induce relatively higher frequencies in the right hemisphere.

## 4 Discussion

In this study, we examined EEG signals from Chen et al. (2024) to explore how observers assess facial trustworthiness during real-time social interactions. Unlike prior research using static facial images, Chen et al. (2024) used live video streams, offering a more ecologically valid perspective. We found that trustworthiness perception is modulated by sustained hemispheric asymmetries, particularly in centroparietal regions from ~800 to 3,000 ms post-stimulus. Whereas earlier studies using static face stimuli suggested that facial trustworthiness perception is rapid and prolonged exposure to facial stimuli did not substantially alter trustworthiness judgments, our findings emphasize the role of dynamic and persistent affective processing in real-time social interactions.

For observers’ behavioral responses, we found that their performance was balanced between trustworthy and untrustworthy conditions, and their accuracy was around the chance level during the time course of the experiment. These findings align with previous research suggesting that judgments of facial trustworthiness can be intuitive and often inaccurate (Bond and DePaulo, 2006; Bond and DePaulo, 2008; Rule et al., 2013; see review by Todorov et al., 2015). One possible explanation is the inherent instability of facial appearance. Prior studies have shown that participants exhibit high variability when identifying faces across different photographs of the same individual, comparable to the variability observed between different individuals (Jenkins et al., 2011; Burton et al., 2016). This high degree of variation has also been observed in social attribution tasks, where judgments about traits like trustworthiness showed similar within- and between-individual variability (Olivola and Todorov, 2010).

Although often inaccurate, research indicates that trustworthiness judgments can be made extremely quickly, within the first 100 ms of stimulus presentation (Willis and Todorov, 2006), a finding supported by EEG studies showing early ERP components reflecting such evaluations (Yang et al., 2011). However, our findings indicate that when viewing faces in real-time, trustworthiness perception is dynamic, as reflected by a sustained interhemispheric phase divergence in the centroparietal cortex. As explained in section 2.4.4, the instantaneous phase represents the position within the cycle of an oscillatory signal at a given time. While phase itself reflects the timing of the waveform, changes in phase over time directly relate to the frequency of the signal: when the phase advances quickly, the frequency is higher; when it advances more slowly, the frequency is lower. This allows us to capture moment-to-moment fluctuations in frequency, even in signals that are nonstationary or not perfectly sinusoidal, which is often the case with EEG data related to brief mental processes (Schack et al., 1999; Kumar et al., 2018; Wang et al., 2006;), rhythmic transitions (Freeman and Rogers, 2002; McIntosh and Sajda, 2020; Nelli et al., 2017), or auditory perception such as speech comprehension (Li et al., 2022) and music perception (Poikonen et al., 2018). In this study, as shown in Figures 6–8, the diverging phase progression between signals at CP1 and CP2 stemmed from a difference in their frequencies, which can be measured by the slope of *conditioning interhemispheric phase shift*, since frequency is defined as the rate of change of unwrapped phase. Further, a spectral centroid analysis was performed to examine how trustworthiness perception impacted the weighted average frequency of signals at CP1 and CP2. As shown in Figure 9, we found that the spectral centroid was higher in trust condition than untrust at CP1, but this pattern reversed at CP2, which indicates a phenomenon of hemispheric lateralization in EEG frequency.

As one of the novelties of the experiment in Chen et al. (2024), using live video streams of players could address two significant limitations associated with the use of posed facial stimuli in conventional emotion recognition research. First, posed facial expressions tend to lack the spontaneity and subtle muscular movements that characterize genuine emotional expressions. Such posed displays are typically categorized or stereotyped in the laboratory conditions and these artificial natures can limit their ecological validity and evoke poorly natural responses from observers (Long et al., 2023; Peluso et al., 2025). As evidence, McLellan et al. (2010) found that observers’ response time to the categorical words was shorter after watching the associated genuine faces than posed faces, which suggests a higher sensitivity to genuine facial expressions than posed ones. In a subsequent fMRI study, McLellan et al. (2012) detected differential cortical activation patterns from observers when they judged posed facial emotion versus genuine facial emotion. However, in this experiment, the players spontaneously generated expressions in response to their internal states and thus the live videos of their facial expressions are more likely to convey authentic, nuanced facial dynamics. This could enhance the realism of the emotional display and provide a richer, more complex set of cues for observers to interpret (Barrett, 2006). Second, static images of faces display only one status of expressions, whereas dynamic stimuli such as live videos provide temporally unfolding information, allowing observers to process the onset and offset of emotional cues, as well as the continuous changes between them. Research suggests that these transitional phases carry important affective information (Filipowicz et al., 2011). Also, it was found that live facial expressions enhanced the observers’ emotional reactions (Hsu et al., 2020). By using live, temporally contiguous expressions, the experiments in this study mirror real-life social interactions more closely, thereby increasing the ecological validity of the experimental setting and improving the generalizability of the findings.

Despite these advantages, the methodology of this experiment also presents certain limitations: it did not record the video feed of the players during the live interaction, nor did they capture eye movement data from the observers. As a result, it is unable to verify precisely which aspects of the facial cues the observers attended to for trustworthiness judgment during the game. Although we detected N170, a typical ERP associated with the perception of emotional faces, from observers’ EEG data, we still could not rule out the possibility that observers may have relied on non-expression cues, such as stable facial structures (Lee et al., 2017), rather than facial expressions alone. Therefore, we cannot conclusively determine the extent to which facial expressions, as opposed to other cues, drove participants’ recognition judgments.

Our key findings are grounded in the well-established phenomenon of hemispheric lateralization. Extensive research has shown that both face and social perception engage lateralized neural networks across the cerebral hemispheres. Early studies highlighted the dominance of the right hemisphere in face processing. For instance, De Renzi (1986) documented two patients with right hemisphere damage who exhibited profound impairments in recognizing familiar faces. Also, Kanwisher et al. (1997) identified the fusiform face area (FFA) as a cortical region specialized for face perception, noting stronger activation in the right hemisphere compared to the left when participants viewed facial stimuli. Subsequent EEG studies further confirmed this lateralization, showing that the face-sensitive ERP component, N170, consistently exhibits greater amplitude in the right hemisphere (Ibáñez et al., 2012; Nakajima et al., 2012; Gao et al., 2019; Dundas et al., 2013, 2014). This right-lateralized N170 effect was also observed in our study, as illustrated in Figures 3, 5. Beyond face perception, the right hemispheric lateralization has also been implicated in processing social information across multiple modalities, including auditory, visual, and olfactory cues (Brancucci et al., 2009). Specifically in the domain of facial stimuli, prior research has shown a perceptual bias toward the left visual field, which was suggested to be associated with right hemisphere processing, in tasks involving gender identification (Butler et al., 2005), assessments of attractiveness (Zaidel et al., 1995), and health judgments (Reis and Zaidel, 2001), further supporting the right hemisphere’s dominancy in social evaluation.

The neural network responsible for face perception is intricately connected to other functional systems, with one particularly important connection being the emotional processing circuitry centered around the amygdala (Said et al., 2010). Emotion-related hemispheric lateralization has been extensively documented within the field of affective neuroscience (see review by Harmon-Jones et al., 2010). Interestingly, emotional face processing has often been associated with left-lateralized amygdala activation. In a comprehensive meta-analysis of PET and fMRI studies, Baas et al. (2004) found that amygdala activation was predominantly lateralized to the left hemisphere when participants were presented with emotional facial stimuli. This pattern of lateralization has also been observed in electrophysiological studies. For instance, early research reported increased alpha activity over the left hemisphere during affective conditions, particularly in posterior brain regions (Smith et al., 1987). Moreover, studies have demonstrated links between left frontal electrical activity and the expression of positive emotions, with greater activation observed during joyful facial expressions (Ekman and Davidson, 1994; Graham and Cabeza, 2001). Supporting these findings, Zotev et al. (2016) used simultaneous fMRI and EEG to show a correlation between frontal EEG asymmetry and amygdala BOLD signal lateralization. Nonetheless, as noted in the Introduction, the precise functional distinctions between the left and right amygdala, as well as the mechanisms linking EEG measures to amygdala activity, remain incompletely understood and warrant further investigation. In our study, we observed hemispheric asymmetry mainly at the centroparietal cortex. Although there is not yet extensive evidence directly linking the centroparietal EEG asymmetry and amygdala activities, simultaneous EEG-fMRI studies have shown that greater amplitudes in centroparietal ERPs co-occurred with increased activation in amygdala and/or its surrounding regions (Sabatinelli et al., 2007; Liu et al., 2012) in emotional processing. This has led to the suggestion that the amygdala may influence the magnitude of the centroparietal EEG activities, possibly through its role in signaling emotional salience to higher-order cortical networks. However, the underlying mechanisms of this interaction remain unclear, and further research is needed to better understand how subcortical emotion processing may be reflected in cortical EEG signals like this.

## Data Availability

The original contributions presented in the study are included in the article/supplementary material, further inquiries can be directed to the corresponding author.
